# Non-Linear Effects of Acute Sleep Deprivation on Spatial Working Memory: Cognitive Depletion and Neural Compensation

**DOI:** 10.3390/brainsci15010018

**Published:** 2024-12-27

**Authors:** Yongcong Shao, Lin Xu, Ziyi Peng, Xin An, Jingjing Gong, Mengfei Han

**Affiliations:** 1 School of Psychology, Beijing Sport University, Beijing 100084, China; budeshao@bsu.edu.cn (Y.S.); rzxulin1997@126.com (L.X.); pzyi121@163.com (Z.P.); axinlll@163.com (X.A.); 2 Laboratory of Sports Stress and Adaptation of General Administration of Sport, Beijing 100084, China; 3 Department of Medical Psychology, Second Medical Center, General Hospital of the People’s Liberation Army, Beijing 100039, China; 4 Aviation Psychology Research Office, Air Force Medical Center, Fourth Military Medical University, Beijing 100142, China

**Keywords:** sleep deprivation, spatial working memory, cognitive depletion, neural compensation, event-related potentials

## Abstract

Background: Spatial working memory is crucial for processing visual and spatial information, serving as a foundation for complex cognitive tasks. However, the effects of prolonged sleep deprivation on its dynamics and underlying neural mechanisms remain unclear. This study aims to investigate the specific trends and neural mechanisms underlying spatial working memory alterations during 36 h of acute sleep deprivation. Methods: Twenty participants underwent a 36 h protocol of acute sleep deprivation. Utilizing the spatial 2-back task for assessing spatial working memory, combined with event-related potential (ERP) technology, we compared behavioral and neural responses at four critical time points—before deprivation, and after 12, 24, and 36 h of sleep deprivation—to uncover dynamic cognitive changes. Results: The findings indicate that the impact of sleep deprivation on spatial working memory exhibits significant temporal dependence. After 24 h of deprivation, both behavioral performance and ERP component amplitudes showed significant declines. During the period from 24 to 36 h, the amplitudes of the P2, N2, and P3 components exhibited a recovery trend, potentially reflecting neural compensatory mechanisms. Conclusions: The impact of 36 h acute sleep deprivation on spatial working memory is characterized by time-dependent and phase-specific effects. Initially, sleep deprivation leads to severe cognitive depletion, followed by an adaptive compensatory phase where neural mechanisms may partially restore function. These findings highlight the non-linear nature of cognitive impairment due to sleep deprivation, involving complex self-regulatory and compensatory mechanisms, with implications for understanding cognitive resilience and adaptive processes.

## 1. Introduction

Working memory, as a core component of the cognitive system, is responsible for the temporary storage and processing of information, serving as the foundation of complex cognitive processes in humans [1]. Among its various forms, spatial working memory specifically involves an individual’s capacity to process and retain visual and spatial information. This ability plays an indispensable role in advanced cognitive tasks such as navigation, target tracking, environmental exploration, and spatial strategy formulation [2,3,4]. Although numerous studies have extensively investigated the effects of various factors—including gender, physical activity, and medication—on spatial working memory [5,6,7], the dynamic changes and underlying neurobiological mechanisms of spatial working memory in extreme situations, particularly under prolonged sleep deprivation, remain insufficiently understood.

Sleep is one of the most fundamental physiological needs for humans, serving not only as a critical factor in the restoration of bodily functions but also as a crucial period for brain memory consolidation and cognitive optimization [8]. However, the fast-paced lifestyle and working patterns of modern society have made sleep deprivation increasingly common, posing a significant public health risk. Prolonged sleep deficiency encroaches upon the valuable time window available for the brain to integrate and optimize memories, while simultaneously impairing cognitive functions and emotional states, such as vigilance, attention, memory capacity, and decision-making efficiency [9,10]. In this context, spatial working memory, as the nexus for processing visual and spatial information, may face unprecedented challenges in sensitivity to information capture, stability in information maintenance, and flexibility in information manipulation, potentially leading to an overall decline in efficiency.

It is noteworthy that current research findings regarding the effects of sleep deprivation on working memory are inconsistent, largely attributed to differences in the duration of sleep restriction. For example, a study by Hennecke et al. [11] reported that reducing sleep to five hours per night for five consecutive nights significantly impaired spatial working memory function. In contrast, Cohen-Zion et al. [12] observed no significant alterations in spatial working memory after four days of six hours of sleep per night, suggesting differences in the impact of varying durations of sleep restriction on cognitive function. Furthermore, research by Peng et al. [13] indicated that after 36 h of extreme sleep deprivation, the brain may compensate for declines in spatial working memory by adjusting interhemispheric dominance, indicating adaptive changes under extreme conditions. Additionally, animal studies [14] have shown that as the duration of sleep deprivation increases, both behavioral performance in spatial working memory and its molecular underpinnings, such as the phosphorylation level of the AMPA receptor subunit GluR1, exhibit cumulative decline. When mice experienced six hours of sleep deprivation, they exhibited a decline in spatial working memory function that did not reach statistical significance. However, with an increase in deprivation duration to twelve hours, significant behavioral impairments and changes in AMPA receptor function were observed, suggesting that the negative effects of sleep deprivation on spatial working memory reached a critical threshold within this timeframe.

These results suggest that spatial working memory may experience both progressive impairment due to mild sleep restriction and immediate impairment resulting from acute sleep deprivation, indicating that the adverse effects of sleep restriction on spatial working memory may vary with its duration. However, there remains a lack of comprehensive research on the specific trajectory of spatial working memory during prolonged sleep deprivation and its accompanying neurophysiological changes. The event-related potential (ERP) technique, with its high temporal resolution, is widely regarded as an ideal method for investigating the impact of sleep deprivation on cognitive brain functions, offering insights into processing states over time [15]. Specifically, the P2 component in ERP, as a marker of early electrical activity, typically emerges within 100–200 milliseconds after stimulus presentation and is closely linked to the rapid recognition, categorization, and initial encoding of visual information, reflecting the brain’s capacity to efficiently capture stimulus features [16,17]. The N2 component represents further cognitive processing, involving information integration, conflict monitoring, and inhibition. Changes in its amplitude directly reflect the brain’s ability to manage cognitive conflicts, make decisions, and suppress irrelevant stimuli [18]. The P3 component, as a critical late potential in ERP studies, provides deeper insights into the strategies for resource allocation and processing efficiency utilized by the brain, closely related to advanced functions such as working memory updating, decision-making, and cognitive control [19,20]. By examining the amplitude variations of the P2, N2, and P3 components, we can evaluate how the brain reallocates resources to sustain or compensate for spatial information processing efficiency during mild to prolonged sleep deprivation.

This study utilizes the spatial n-back paradigm as a testing task for assessing spatial working memory, combined with event-related potential (ERP) technology. By comparing behavioral data and ERP data at four critical time points—prior to sleep deprivation and after 12, 24, and 36 h of sleep deprivation—this research aims to examine specific trends in spatial working memory changes during 36 h of acute sleep deprivation and the associated neural mechanisms. Specifically, this study posits the following hypotheses: (1) As the duration of continuous sleep deprivation increases, performance in spatial working memory (accuracy and reaction time) is expected to show a significant decline, especially after 24 h, where the degree of impairment is anticipated to increase considerably. (2) The ERP components P2, N2, and P3 are expected to exhibit specific patterns of change during sleep deprivation. Amplitude decreases in P2 and N2 are anticipated in early stages, indicating reduced primary visual processing and conflict monitoring; the P3 amplitude is expected to decrease significantly during prolonged deprivation, suggesting declines in higher-order cognitive control and working memory updating. This study aims to advance understanding of how sleep deprivation impacts spatial working memory, addressing the gap in research on its dynamic changes and neural mechanisms during prolonged deprivation, while offering new insights and approaches to mitigate cognitive declines linked to sleep deprivation.

## 2. Materials and Methods

### 2.1. Participants

This study employed a convenience sampling method to recruit university student participants who met the inclusion and exclusion criteria. Sample size estimation was conducted using G*Power 3.1.9 software, with the following parameters: effect size f = 0.25, α = 0.05, power = 0.8, number of groups = 1, and number of measurements = 4. The calculation indicated that 24 participants were required to achieve the target power. However, due to the specificity of the 36 h sleep deprivation experiment, the actual recruitment resources for this study were limited, and we recruited 20 participants (14 males and 6 females) to participate in the experiment. Detailed demographic information about the participants can be found in Table 1. The inclusion criteria for participants were as follows: (1) normal vision or corrected to normal; (2) no physical illnesses, psychological disorders, or sleep-related issues; (3) participants were screened using the Pittsburgh Sleep Quality Index (PSQI) to ensure that they had good sleep hygiene and a normal sleep–wake cycle, adhering to a regular sleep schedule of going to bed between 10 PM and 11 PM and waking up between 7 AM and 8 AM, with a minimum of 8 h of sleep per night; (4) a score of IQ > 110 on the Raven’s Standard Progressive Matrices; (5) a score of ≥110 on the Clinical Memory Scale Test; (6) a score within the normal range on the Beck Depression Inventory. The exclusion criteria were as follows: (1) a history of or current diagnosis of sleep disorders, anxiety, depression, or other psychological disorders; (2) current smokers; (3) recent use of medications (such as sleeping pills, antidepressants, anxiolytics, sedatives, etc.); (4) addiction to caffeine, tea, or alcoholic beverages; (5) participation in other interfering experiments. Participants received a detailed explanation of the sleep deprivation process, potential risks, study objectives, and procedures, and provided written informed consent. The study strictly adhered to ethical research principles, with formal approval of the experimental protocol from the Ethics Committee of Beihang University. Two participants discontinued their participation during the sleep deprivation phase, while the behavioral and ERP data from the remaining 18 participants were included in the final data analysis.

### 2.2. Experimental Design

This study employed a single-factor, four-level repeated-measures design (sleep deprivation duration: 0, 12, 24, and 36 h) to investigate the dynamic changes in spatial working memory under acute sleep deprivation. The experiment was conducted in the psychology laboratory of Beijing Sport University. Data were collected through laboratory-based behavioral tests and ERP recordings. During the experiment, environmental conditions (e.g., lighting and temperature) were rigorously controlled to ensure the accuracy and consistency of the data.

### 2.3. Experimental Task

This study employs the classic spatial 2-back working memory paradigm to assess individuals’ capacity to maintain and update spatial information in complex, dynamic environments. During the experiment, participants were required to monitor the position of each stimulus (i.e., black squares) appearing randomly on a 3 × 3 grid, determining quickly and accurately whether each new stimulus matched the stimulus presented two positions previously. The experiment employed E-Prime 2.0 to precisely control the sequence, position, and duration of each stimulus presentation, ensuring standardization and consistency of experimental conditions.

**Figure 1:**
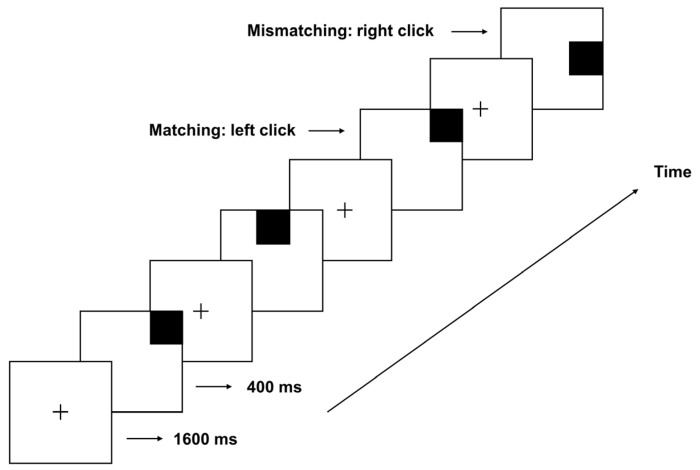
Schematic of the spatial 2-back task. Participants monitored sequential black squares on a 3 × 3 grid, indicating whether each square matched the position from two trials prior (left click for match, right click for mismatch). Each trial began with a 1600 ms fixation (indicated by a “+”) followed by a 400 ms stimulus. Matching and non-matching trials were presented in a 1:1 ratio.

At the beginning of each trial, a fixation point was presented at the center of the screen for 1600 ms, indicating an upcoming stimulus. The first black square then appeared at a random grid position (e.g., position A) for 400 ms before disappearing. Another fixation point was subsequently displayed for 1600 ms, allowing participants to consolidate memory and prepare for the upcoming stimulus. The second black square (position B) was then presented similarly (displayed for 400 ms before disappearing), requiring participants to retain positions A and B in memory. Upon appearance of the third black square (position C), participants were tasked with determining quickly and accurately whether its position matched that of position A. If the positions matched, participants pressed the left mouse button; if not, they pressed the right mouse button. Trials with matching and non-matching positions were randomly mixed in a 1:1 ratio to balance experimental conditions. A practice task was administered before the formal experiment to ensure data reliability, requiring participants to attain a minimum accuracy rate of 85% before proceeding (Figure 1).

### 2.4. Experimental Procedure

**Figure 2:**
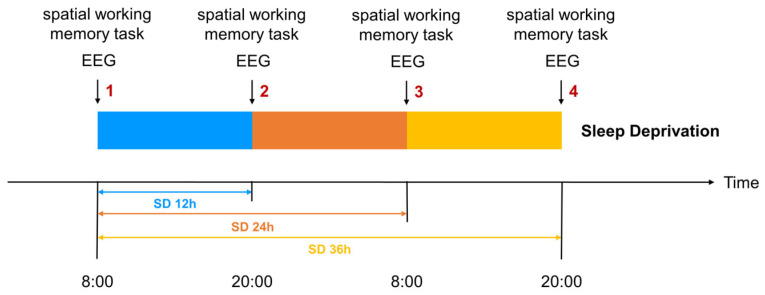
The experimental arrangement and data collection timeline during the 36 h sleep deprivation protocol. Behavioral and EEG data were collected at baseline (0 h) and after 12, 24, and 36 h of sleep deprivation (1, 2, 3, 4 in Figs. moment points), with spatial working memory tasks administered at each time point.

Participants who met the recruitment criteria entered the laboratory at 6 PM the evening before the experiment to ensure each participant received 7–8 h of adequate sleep, eliminating potential sleep debt and establishing a baseline for the subsequent sleep deprivation phase. Participants naturally awakened at 8 AM the following morning and completed the first spatial working memory task, marking the start of the 36 h sleep deprivation period. This time point was designated as the 0 h mark for sleep deprivation, which ended at 8 PM. the next day. Data collection occurred at four key time points: baseline (0 h), 12 h, 24 h, and 36 h. At each time point, participants completed behavioral assessments using the spatial n-back task and event-related potential (ERP) recordings to evaluate spatial working memory performance and its associated neural activity (Figure 2). The 12 h time point was chosen to represent a typical waking retention interval. This time point serves as a critical baseline to observe potential transitional changes en route to prolonged sleep deprivation.

Participants were required to remain awake for the following 36 h. To ensure adherence, several measures were implemented: (1) Environmental control: the laboratory maintained moderate lighting and temperature to prevent conditions that might induce sleep. (2) Supervision and interaction: the experimenters engaged in brief interactions with the participants every two hours, requiring them to subjectively report their current fatigue levels while observing their psychological state and documenting any abnormal behaviors. If significant psychological abnormalities or discomfort were observed, the experiment was immediately terminated, and appropriate support was provided. (3) Leisure activities: participants were allowed to engage in non-stimulating recreational activities, including reading, watching light films, and listening to soft music, to alleviate monotony and fatigue. (4) Dietary management: nutritionally balanced meals were provided at regular intervals to maintain participants’ energy during sleep deprivation. To avoid interference with results, intake of stimulant-containing substances (caffeine, tea, cola, or any caffeinated medications) was strictly prohibited, and compliance was verified through interviews and dietary monitoring. (5) Safety monitoring was rigorously implemented throughout the study, with continuous assessments of participants’ physiological and psychological states and routine inquiries into their subjective well-being. In the event of any health risks or discomfort, immediate measures were taken, including the potential termination of the experiment.

### 2.5. ERP Data Acquisition

The Neuroscan EEG acquisition system and a 32-channel Ag/AgCl electrode cap were employed to record online EEG data, with electrode placement conforming to the International 10-5 System. HEOG electrodes were positioned laterally on both eyes, while VEOG electrodes were placed above and below the left eye. The SynAmps2 amplifier (Compumedics Neuroscan, Charlotte, NC, USA) was used to amplify the recorded signals, with bilateral mastoids serving as reference electrodes. HEOG electrodes were positioned approximately 1 cm from the outer canthi of both eyes, and VEOG electrodes were positioned 1.5 cm above and below the left eye. Electrode-to-scalp impedance was maintained below 5 kΩ. Offline EEG data analysis was performed using the EEGLAB [21] and ERPLAB [22] toolboxes in MATLAB 2018b (MathWorks, Natick, MA, USA). The EEG data were first downsampled to 500 Hz, then re-referenced to the average, and filtered with a band-pass range of 0.1 to 30 Hz. Artifacts were manually removed, followed by independent component analysis (ICA) to correct components associated with movement and blink artifacts. Additional artifact correction was applied to remove EEG segments exceeding ±100 µV.

### 2.6. Data Statistical Analysis

#### 2.6.1. Behavioral Data Analysis

Behavioral data were thoroughly analyzed using SPSS version 27.0. One-way repeated measures ANOVA (RM-ANOVA) was performed to analyze reaction times and accuracy, with sleep deprivation duration (0, 12, 24, and 36 h) as the independent variable. When the assumption of sphericity was violated, as determined by Mauchly’s test, we employed Multivariate Analysis of Variance (MANOVA) to adjust the results. This approach accounts for the potential intercorrelation among dependent variables and provides robust results when the sphericity assumption is violated in the RM-ANOVA. The Least Significant Difference (LSD) method was applied to post hoc comparisons of main effects to examine significant differences in reaction times and accuracy across sleep deprivation conditions.

#### 2.6.2. ERP Data Analysis

The 200 ms period preceding stimulus onset was used as the baseline, with analysis covering the 200 ms pre-stimulus to 800 ms post-stimulus window. The electrode selection was guided by well-established ERP research. Frontal electrodes (Fz, F4, FC3, FCz, FC4) are known for their sensitivity to early cognitive processes such as visual recognition and conflict monitoring, as reflected in the P2 (150–200 ms) and N2 (200–300 ms) components [13,23]. Parietal electrodes (Cz, C4, CP3, CPz, CP4) were chosen for the P3 (300–500 ms) component, which is associated with higher-order cognitive processes like working memory updating and decision-making [19,24]. One-way repeated measures ANOVA (RM-ANOVA) was conducted separately for the P2, N2, and P3 components (sleep deprivation durations: 0 h, 12 h, 24 h, 36 h). MANOVA was used to adjust results when sphericity was violated, with post hoc main effect comparisons corrected using the LSD method.

## 3. Results

### 3.1. Behavioral Results

**Figure 3:**
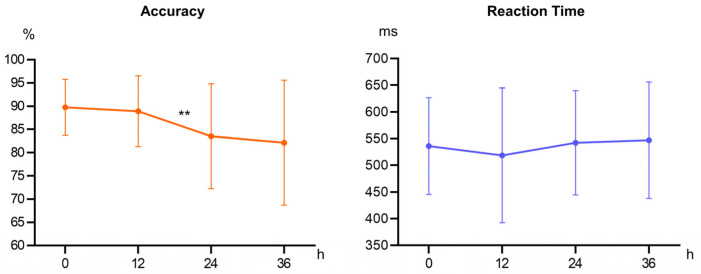
Behavioral results of accuracy and reaction time across different sleep deprivation durations (0, 12, 24, 36 h). Accuracy declined significantly between 12 and 24 h (** means *p* < 0.01).

The analysis of accuracy revealed a significant main effect of sleep deprivation duration (*F*_(3, 15)_ = 3.602, *p* = 0.039, η^2^_p_ = 0.419). During the initial phase of sleep deprivation (within the first 12 h), participants’ accuracy remained relatively high without significant difference from baseline levels (*p* = 0.330), likely reflecting their ability to operate within a typical waking retention interval. This suggests that the 12 h condition may primarily capture baseline cognitive performance under normal waking conditions. However, as sleep deprivation duration increased, a significant decline in accuracy was observed between 12 and 24 h (*p* = 0.010). This transition from 12 to 24 h may represent a critical turning point where sleep deprivation substantially impairs cognitive function. Although a further decrease in accuracy was noted after 36 h compared to 24 h, this change did not attain statistical significance (*p* = 0.322), indicating that the rate of cognitive impairment may have slowed or stabilized after 24 h of continuous deprivation. The analysis of reaction times demonstrated a non-significant main effect of sleep deprivation duration (*F*_(3, 51)_ = 0.885, *p* = 0.455, η^2^_p_ = 0.049) (Table 2, Figure 3).

### 3.2. ERP Results

**Figure 4:**
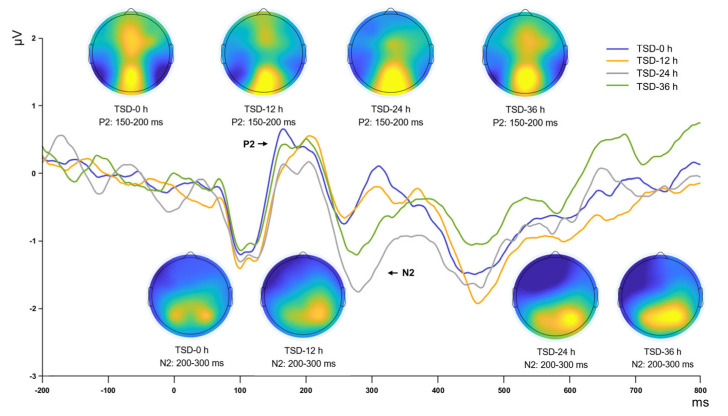
ERP waveforms and topographical maps showing the P2 (150–200 ms) and N2 (200–300 ms) components across different sleep deprivation durations (0, 12, 24, and 36 h).

Statistical analysis of the P2 component revealed a significant main effect of sleep deprivation duration (*F*_(3, 15)_ = 7.188, *p* = 0.003, η^2^_p_ = 0.590). Further post hoc comparisons demonstrated that during the first 12 h of sleep deprivation, the amplitude of the P2 component did not significantly decrease (*p* = 0.156), suggesting that the P2 amplitude remained relatively stable during this period. However, with the duration of deprivation extending to 24 h, the amplitude of the P2 component exhibited a highly significant reduction compared to baseline levels (*p* < 0.001), indicating a substantial impact of prolonged sleep loss on early stages of information processing in the brain. Notably, this decreasing trend in amplitude did not further intensify. When deprivation reached 36 h, although the P2 amplitude partially recovered compared to the 24 h low, this change did not reach statistical significance (*p* = 0.059), suggesting that some physiological or cognitive mechanisms may be at play, enabling the brain to attempt to maintain a level of functional stability under extreme fatigue conditions (Table 3, Figure 4).

Statistical analysis of the N2 component revealed a significant main effect of the duration of sleep deprivation (*F*_(3, 15)_ = 3.611, *p* = 0.038, η^2^_p_ = 0.419). Post hoc comparisons demonstrated a detailed pattern of changes in N2 amplitude over time: during the first 12 h of sleep deprivation, the N2 amplitude remained stable, without significant changes (*p* = 0.807), suggesting that the N2 component may not have been significantly affected during this period. However, as the duration of deprivation increased, a sharp decline in N2 amplitude was evident between 12 and 24 h (*p* = 0.005), indicating the sensitivity of the N2 component to prolonged sleep deprivation. When the duration of deprivation was extended from 24 to 36 h, the N2 amplitude change pattern mirrored the previously observed P2 component, with an amplitude rebound compared to the 24 h mark (*p* = 0.034). This rebound may reflect a compensatory or adaptive adjustment by the participants following extended sleep deprivation (Table 3, Figure 4).

**Figure 5:**
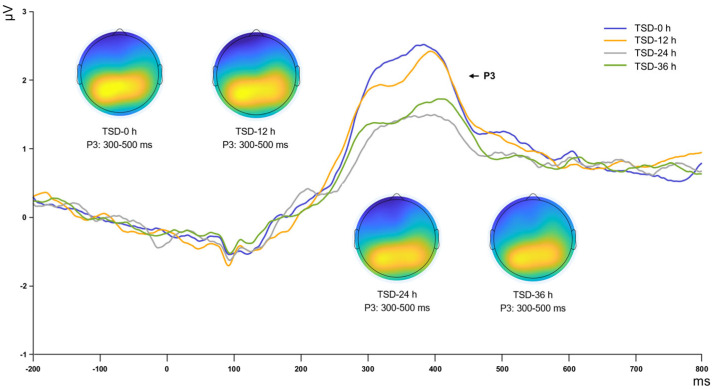
ERP waveforms and topographical maps showing the P3 (300–500 ms) components across different sleep deprivation durations (0, 12, 24, and 36 h).

Statistical analysis of the P3 component revealed a significant main effect of the duration of sleep deprivation (*F*_(3, 15)_ = 3.358, *p* = 0.047, η^2^_p_ = 0.402). Post hoc comparisons showed that during the first 12 h of sleep deprivation, the amplitude of the P3 component remained highly stable, with no significant changes observed (*p* = 0.846), suggesting that participants were able to maintain relatively stable resource allocation and memory updating abilities. However, as deprivation duration increased, a sharp decline in P3 amplitude occurred between 12 and 24 h (*p* = 0.010), reflecting significant impairment in the brain’s encoding and storage capabilities for spatial information during this phase. When deprivation was further extended to 36 h, the P3 amplitude neither continued to deteriorate nor showed the anticipated rebound but remained at a level similar to that at 24 h (*p* = 0.835) (Table 3, Figure 5).

## 4. Discussion

Sleep is essential for maintaining normal cognitive function and is particularly critical for spatial working memory, a process involving complex cognitive tasks. This study systematically examined the effects of 36 h of acute sleep deprivation on spatial working memory and its underlying neural mechanisms through the spatial n-back paradigm and ERP technology. Our findings revealed a nonlinear pattern of changes in spatial working memory performance attributable to sleep deprivation, accompanied by phase-specific neural response adjustments.

The impact of sleep deprivation on participants’ accuracy during the initial 12 h was limited, with accuracy remaining high. This suggests that the 12 h time point reflects typical cognitive functioning within a normal waking interval, where sleep deprivation effects are not yet evident. Therefore, the observed performance stability likely represents baseline cognitive capabilities [25]. After 24 h, participants’ accuracy declined significantly, indicating that the depletion of cognitive resources made it challenging to meet task demands. Studies show that the negative effects of sleep deprivation emerge after one night of insufficient sleep and worsen with extended wakefulness, as the brain cannot effectively mobilize attention and memory resources, leading to a noticeable decline in cognitive performance [26,27]. Following 36 h, the decline in accuracy plateaued, suggesting that the brain may activate self-regulatory mechanisms to maintain cognitive function, possibly reallocating remaining cognitive resources or activating alternative neural networks. Subsequent ERP results support this compensatory mechanism. Reaction time data showed no significant differences across time points, possibly due to strategic adjustments when cognitive resources are limited. Individuals may maintain stable reaction times at the expense of accuracy when resources are scarce [28].

From a neurophysiological perspective, the changes observed in the P2, N2, and P3 ERP components collectively illuminate the impact of sleep deprivation on cognitive functions, such as early sensory information processing, conflict monitoring, and attentional resource allocation [19,29,30]. Following 12 h of continuous sleep deprivation, the amplitudes of these components remained stable, suggesting that initial perception, attentional orientation, conflict monitoring, and working memory updating were not significantly impacted in the short term. With sleep deprivation extended to 24 h, a marked decline in the amplitudes of the P2, N2, and P3 components was observed. The substantial decrease in P2 amplitude suggests a deterioration in the brain’s resource allocation at the early sensory processing level, potentially impairing selective attention. Similarly, the decline in N2 amplitude indicates weakened conflict monitoring abilities, possibly resulting in difficulties making accurate decisions. Moreover, the reduction in P3 amplitude reflects a marked decrease in the brain’s ability to process new information and allocate cognitive resources, potentially leading to reduced accuracy and increased fatigue during complex tasks. These effects may result from the sustained burden of sleep deprivation on neural networks, reducing the efficiency of perceptual processing in the sensory cortex [31,32].

After 36 h of sleep deprivation, an increase in the amplitudes of the P2, N2, and P3 components was observed, characterized as a rebound trend. Among these, the N2 trend was statistically significant and the P2 trend was marginally significant, whereas the P3 effect did not reach statistical significance. These findings suggest that the brain may employ adaptive mechanisms to mitigate cognitive impairment, although further studies with larger samples are needed to confirm this hypothesis. Research by Drummond et al. [33] suggests these strategies may entail the activation of additional brain regions or the formation of compensatory neural networks. Recent findings corroborate this observation: Tian et al. [34] demonstrated that after sleep deprivation, the brain reallocates cognitive resources by enhancing connectivity in hub regions, particularly within control and significant networks, reflecting a compensatory mechanism. This finding also suggests an adaptive response of the brain to environmental demands. Chengyang et al. [35] and Yoo et al. [36] noted significant enhancements in thalamus-precuneus connectivity under sleep deprivation, indicating notable recovery in working memory performance despite fatigue. This compensatory mechanism facilitates the partial restoration of key behavioral performance during sleep deprivation, demonstrating the brain’s adaptability and resilience under extreme conditions. However, this compensation is not without limits. The dynamic changes in the amplitudes of the P2, N2, and P3 components during 36 h of sleep deprivation reveal a two-phase brain response mechanism when confronted with cognitive resource depletion and fatigue, involving neural network reactivation or cognitive strategy alterations to minimize cognitive function loss.

The pattern of amplitude variation is not solely a consequence of sleep deprivation but may also be significantly affected by circadian rhythms. Circadian rhythms, regulated by the brain’s internal biological clock, significantly affect cognitive performance throughout the day [37,38,39,40]. It is traditionally believed that cognitive and neural functions peak in the morning, which is associated with increased arousal and attention. Nevertheless, studies indicate that circadian rhythms do not consistently predict optimal memory performance time, with many species exhibiting unclear correlations between memory formation rhythms and activity rhythms [38]. Following 24 h of sleep deprivation, although individuals are theoretically at physiological arousal peak in the morning, extensive sleep loss resulted in significant cognitive resource depletion and a notable decline in performance and neural activity. This suggests that fatigue’s cumulative effects can negate circadian rhythms’ facilitative influence on cognitive function, even during favorable periods. At 36 h, as circadian rhythms reach a trough, individuals may experience deeper fatigue, yet the brain may concurrently initiate complex compensatory mechanisms to mobilize remaining resources, partially restoring neural activity and maintaining cognitive functions. This interplay between circadian rhythms and sleep deprivation reveals mechanisms by which the brain sustains cognitive functions under extreme fatigue, offering new research directions and perspectives.

Despite revealing a significant impact of 36 h sleep deprivation on the temporal evolution of spatial working memory, this study presents several limitations. (1) The variability in individual sensitivity to sleep deprivation may have substantially influenced the observed results, particularly the changes in ERP amplitude and behavioral outcomes. While the limited sample size constrained our ability to completely disentangle the underlying effects, analyses of standard deviations offer preliminary indications of these differences. Future studies should incorporate larger sample sizes and include individual difference variables, such as gender, genetic predispositions, and chronotype assessments. Additionally, adopting mixed-effects models or hierarchical Bayesian analyses will enable a more rigorous accounting of inter-individual variability, enhancing generalizability and fostering a deeper understanding of these differences. (2) This study focused on acute sleep deprivation without addressing the effects of long-term chronic sleep deficiency on cognitive function. The mechanisms through which acute and chronic sleep deprivation affect the brain may differ; future studies should investigate the long-term effects on brain function using paradigms imposing chronic sleep restrictions. (3) This research employed the spatial n-back task as the main cognitive task; future studies could validate the effects of sleep deprivation across various cognitive processes by incorporating diverse working memory tasks (e.g., verbal or object working memory tasks) to develop a more comprehensive cognitive function model. (4) After 36 h of sleep deprivation, all participants underwent an 8 h recovery sleep in the laboratory to restore their normal state. However, as this study primarily focused on the direct effects of sleep deprivation on cognitive function, we regret that no further cognitive assessments were conducted following the recovery sleep phase. To address this limitation, future research will include the assessment of participants’ cognitive function across the full cycle from sleep deprivation to recovery sleep. This will provide a more comprehensive understanding of the dynamic changes in cognitive performance associated with sleep loss and recovery.

## 5. Conclusions

This study employed the spatial n-back task with ERP technology to thoroughly investigate the effects of 36 h of continuous acute sleep deprivation on spatial working memory and its underlying neural mechanisms. The results demonstrated that the impact of sleep deprivation on spatial working memory exhibited significant temporal dependence and phase-specific characteristics. After 24 h of sleep deprivation, both behavioral performance and the amplitudes of relevant ERP components experienced significant declines, indicating a severe depletion of cognitive resources during this phase. While overall cognitive function remained impaired from 24 to 36 h, the P2, N2, and P3 components exhibited distinct recovery patterns. Specifically, the recovery of P2 amplitude suggests an improvement in early sensory processing and attentional allocation; the rebound in N2 amplitude indicates enhanced conflict monitoring and cognitive control; and the limited recovery of P3 amplitude reflects the continued constraint on higher-order cognitive functions. These findings highlight that cognitive impairment caused by sleep deprivation is not a simple linear process but rather involves complex, component-specific self-regulatory and compensatory mechanisms.

## Figures and Tables

**Table 1:** Demographic information of participants.

Variable	Value
Participants Number	20
Gender Distribution	14 males, 6 females
Mean Age (SD)	21.925 ± 1.184
Education Level	University students
Occupational Status	Full-time student

**Table 2:** Mean and standard deviation of reaction times and accuracy at baseline (0 h) and after 12, 24, and 36 h of sleep deprivation.

	Response Time (ms)	Accuracy (%)
TSD-0 h	536.107 ± 90.933	89.745 ± 6.021
TSD-12 h	518.384 ± 126.445	88.889 ± 7.613
TSD-24 h	542.044 ± 98.004	83.522 ± 11.311
TSD-36 h	546.843 ± 109.154	82.156 ± 13.455

**Table 3:** Mean and standard deviation of P2, N2, and P3 amplitude at baseline (0 h) and after 12, 24, and 36 h of sleep deprivation.

	P2 (μV)	N2 (μV)	P3 (μV)
TSD-0 h	1.382 ± 1.791	−1.449 ± 2.628	3.296 ± 1.611
TSD-12 h	1.022 ± 1.730	−1.321 ± 2.274	3.248 ± 1.450
TSD-24 h	0.681 ± 1.829	−2.475 ± 2.664	2.332 ± 1.565
TSD-36 h	1.346 ± 1.713	−1.743 ± 2.709	2.387 ± 1.520

## Data Availability

The data presented in this study are available on request from the corresponding author due to commitments made in the informed consent to protect participant privacy.
